# Supplementary material for: DynaMight: estimating molecular motions with improved reconstruction from cryo-EM images
Source: Nat Methods. 2024 Aug 9;21(10):1855–62. doi: 10.1038/s41592-024-02377-5 (PMC11466895; doi:10.1038/s41592-024-02377-5)
Supplement: Supplementary file 1 — Supplementary Table 1, Fig. 1 and Legends for Videos 1–6. [file 41592_2024_2377_MOESM1_ESM.pdf]

nature methods

Supplementary information https://doi.org/10.1038/s41592-024-02377-5

DynaMight: estimating molecular motions with improved reconstruction from cryo-EM images

In the format provided by the authors and unedited

Supplementary information to: DynaMight: estimating molecular motions with improved reconstruction from cryo-EM images

Johannes Schwab[1,*], Dari Kimanius[1,2], Alister Burt[1,3], Tom Dendooven[1], Sjors H.W. Scheres[1,*]

[1] MRC Laboratory of Molecular Biology,
Francis Crick Avenue, Cambridge, CB2 0QH, UK
[2] CZ Imaging Institute,
3400 Bridge Parkway, Redwood City, CA 94065, USA
[3] Department of Structural Biology, Genentech, South San Francisco
CA 94080, USA
Cambridge, MA 02142, USA
[*] Correspondence to {schwab,scheres}@mrc-lmb.cam.ac.uk

	Spliceosome	tri-snRNP	CCAN:CENP-A	Kinetochore
**EMPIAR**	10180	10073	11910	11890
**EMDB-id**	EMD-19791	EMD-19789	EMD-19799	EMD-19794
**nr. particles**	44,537	86,624	100,311	108,672
**box size (pixel)**	320	380	360	320
**pixel size (Å)**	1.699	1.4	0.853	1.08
**nr. gaussians**	30,000	30,000	30,000	30,000
**nr. epochs**	221	273	373	290
**nr. latent dimensions**	5	10	10	10
**compute time (h)**	9+6+4	24+7+16	17+8+20	27+11+24
**GPU used**	A6000	A6000	A100	A100
**mean deformations (Å)**	7.755	4.303	3.608	4.112
**max deformation (Å)**	24.139	14.071	8.248	8.802

Supplementary Table 1: Details of the four data sets. Approximate compute times are given in hours (h) for the estimation of the deformations, the estimation of the inverse deformations, and the calculation of the improved reconstruction, respectively. The mean and maximum deformations for all particles in a data set is calculated as the root mean square deviation between the Gaussian positions in the reference and the displaced Gaussians for each particle.

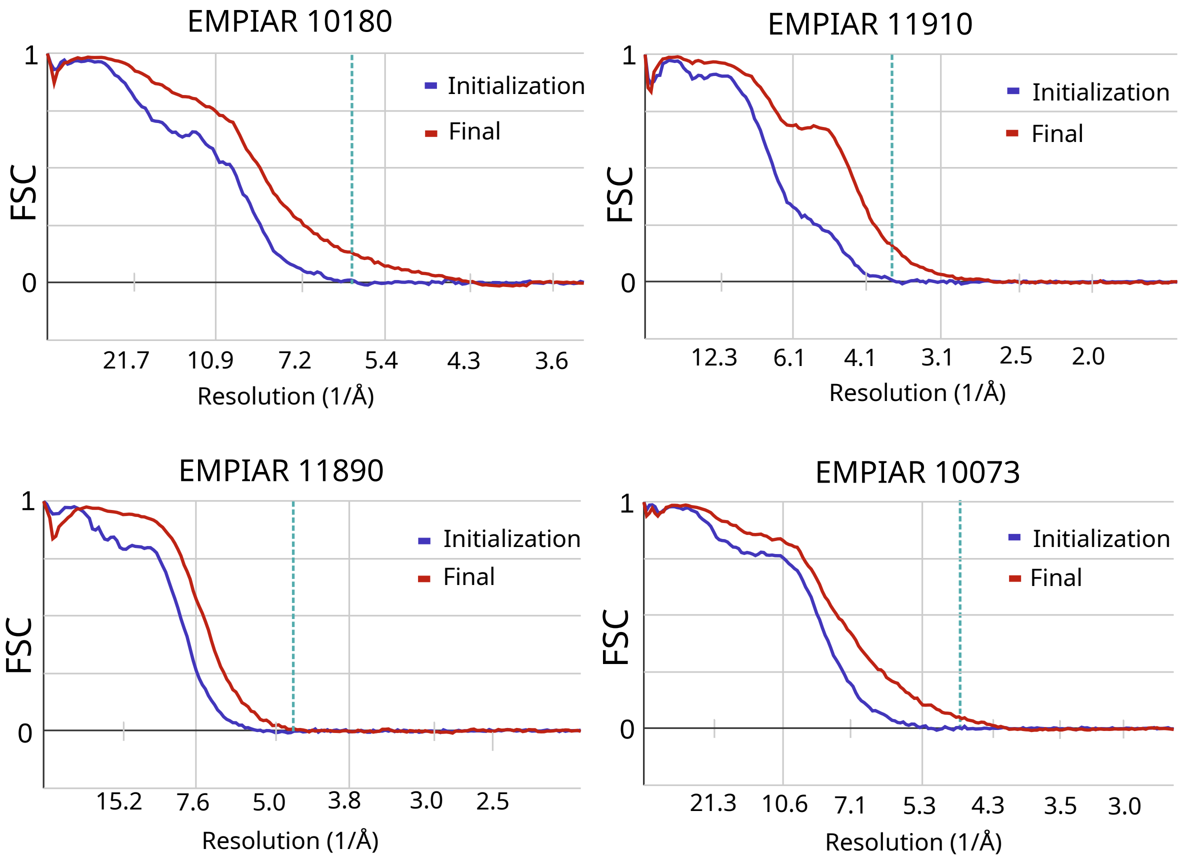

Supplementary Figure 1: Fourier shell correlation (FSC) Gaussian model. FSC between the Gaussian consensus model, of half set 1, and the reconstructed map from the consensus refinement (blue) and between the final Gaussian model and the improved DynaMight reconstruction (red). The dashed cyan line indicates the reported resolution of the masked consensus refinement.

Supplementary Video 1: DynaMight reconstruction using incorrect prior for SF3a region. The video shows the DynaMight reconstructions with deformations obtained with the prior used from the correct atomic model (right) and the atomic model with incorrect SF3a domain (left). The reconstructed density with the wrong prior clearly resembles the incorrect model.

Supplementary Video 2: DynaMight reconstruction using incorrect prior for SF3b region. The video shows the DynaMight reconstructions with deformations obtained with the prior used from the correct atomic model (right) and the atomic model with incorrect SF3b domain (left). For this better resolved region, the resemblance with the incorrect model is weaker than in Supplementary Data Video 1.

Supplementary Video 3: Observed motions for the pre-catalytic spliceosome complex. The video shows a trajectory through latent space of half 1 for a subset of the EMPIAR-10180 data set. The trajectory was chosen such that it covers disparate regions in latent space.

Supplementary Video 4: Observed motions for the spliceosomal tri-snRNP complex. The video shows a trajectory through latent space of half 1 for the 86,624 selected particles of the EMPIAR-10073 data set. The trajectory was chosen such that it disparate regions in latent space.

Supplementary Video 5: Observed motions for the CCAN:CENP-A complex. The video shows a trajectory through latent space of half 1 for the EMPIAR-11910 data set. The trajectory was chosen such that it covers disparate regions in latent space.

Supplementary Video 6: Observed motions of the complete inner kinetochore complex. The video shows a trajectory through latent space of half 1 for the EMPIAR-11890 data set. The trajectory was chosen such that it covers disparate regions in latent space
