# Supplementary material for: DynaMight: estimating molecular motions with improved reconstruction from cryo-EM images
Source: Nat Methods. 2024 Aug 9;21(10):1855–62. doi: 10.1038/s41592-024-02377-5 (PMC11466895; doi:10.1038/s41592-024-02377-5)
Supplement: Supplementary file 2 — Reporting Summary [file 41592_2024_2377_MOESM2_ESM.pdf]

nature portfolio

Corresponding author(s): Sjors Scheres

Last updated by author(s): 2024/05/27

Reporting Summary

Nature Portfolio wishes to improve the reproducibility of the work that we publish. This form provides structure for consistency and transparency in reporting. For further information on Nature Portfolio policies, see our Editorial Policies and the Editorial Policy Checklist.

Statistics

For all statistical analyses, confirm that the following items are present in the figure legend, table legend, main text, or Methods section.

n/a	Confirmed	
☐	☒	The exact sample size (*n*) for each experimental group/condition, given as a discrete number and unit of measurement
☒	☐	A statement on whether measurements were taken from distinct samples or whether the same sample was measured repeatedly
☒	☐	The statistical test(s) used AND whether they are one- or two-sided<br>*Only common tests should be described solely by name; describe more complex techniques in the Methods section.*
☒	☐	A description of all covariates tested
☒	☐	A description of any assumptions or corrections, such as tests of normality and adjustment for multiple comparisons
☐	☒	A full description of the statistical parameters including central tendency (e.g. means) or other basic estimates (e.g. regression coefficient) AND variation (e.g. standard deviation) or associated estimates of uncertainty (e.g. confidence intervals)
☒	☐	For null hypothesis testing, the test statistic (e.g. *F*, *t*, *r*) with confidence intervals, effect sizes, degrees of freedom and *P* value noted<br>*Give P values as exact values whenever suitable.*
☒	☐	For Bayesian analysis, information on the choice of priors and Markov chain Monte Carlo settings
☒	☐	For hierarchical and complex designs, identification of the appropriate level for tests and full reporting of outcomes
☒	☐	Estimates of effect sizes (e.g. Cohen's *d*, Pearson's *r*), indicating how they were calculated

Our web collection on statistics for biologists contains articles on many of the points above.

Software and code

Policy information about availability of computer code

Data collection	Data collection software is not relevant to this study
Data analysis	DynaMight is distributed for free under a BSD license and can be downloaded from https://github.com/3dem/DynaMight. It is installed automatically with RELION-5.0

For manuscripts utilizing custom algorithms or software that are central to the research but not yet described in published literature, software must be made available to editors and reviewers. We strongly encourage code deposition in a community repository (e.g. GitHub). See the Nature Portfolio guidelines for submitting code & software for further information.

Data

Policy information about availability of data

All manuscripts must include a data availability statement. This statement should provide the following information, where applicable:

- Accession codes, unique identifiers, or web links for publicly available datasets
- A description of any restrictions on data availability
- For clinical datasets or third party data, please ensure that the statement adheres to our policy

The authors declare that there are no restrictions on data availability. The data sets of the CCAN:CENP-A complex and of the yeast inner kinetochore complex bound to a CENP-A have been deposited at EMPIAR and are available under accession codes 11890 and 10073, respectively. Data sets of the spliceosome complexes were already available under accession codes 10180 and 10073. Atomic coordinates from PDB entries 5nrl, 1g88 and 7yuy were also used in this study. The local

resolution filtered reconstructions and the DynaMight half-maps for all datasets are available on EMDB under the accession codes EMD-19791 for the pre-catalytic spliceosome, EMD-19789 for the tri-snRNP complex, EMD-19799 for the CCAN:CENP-A complex and EMD-19794 for the yeast inner kinetochore complex bound to CENP-A.

Human research participants

Policy information about studies involving human research participants and Sex and Gender in Research.

Reporting on sex and gender	N/A
Population characteristics	N/A
Recruitment	N/A
Ethics oversight	N/A

Note that full information on the approval of the study protocol must also be provided in the manuscript.

Field-specific reporting

Please select the one below that is the best fit for your research. If you are not sure, read the appropriate sections before making your selection.

☒ Life sciences ☐ Behavioural & social sciences ☐ Ecological, evolutionary & environmental sciences

For a reference copy of the document with all sections, see nature.com/documents/nr-reporting-summary-flat.pdf

Life sciences study design

All studies must disclose on these points even when the disclosure is negative.

Sample size	Cryo-EM data set sizes were determined by the publicly available data sets or the experimentalists providing the data. They were originally probably determined by the amount of available microscopy time. The number of data sets described in this study was deemed sufficient to illustrate the general usefulness of the DynaMight approach.
Data exclusions	We selected a subset of 45k spliceosome particles from the EMPIAR-10180 data set in order to make it compositionally homogeneous. We used standard 3D classification in RELION to make this selection, as DynaMight cannot handle structural heterogeneous data sets.
Replication	No replication experiments were performed, as noise on the outcome of the computational analysis was not considered to be affecting the conclusions.
Randomization	For each data set, two random half-sets were employed for resolution estimation. Randomisation was performed using a random number generator.
Blinding	No blinding was performed as bias from the user is not expected to affect the outcome of the computational analysis.

Reporting for specific materials, systems and methods

We require information from authors about some types of materials, experimental systems and methods used in many studies. Here, indicate whether each material, system or method listed is relevant to your study. If you are not sure if a list item applies to your research, read the appropriate section before selecting a response.

Materials & experimental systems

n/a	Involved in the study	
☒	☐	Antibodies
☒	☐	Eukaryotic cell lines
☒	☐	Palaeontology and archaeology
☒	☐	Animals and other organisms
☒	☐	Clinical data
☒	☐	Dual use research of concern

Methods

n/a	Involved in the study	
☒	☐	ChIP-seq
☒	☐	Flow cytometry
☒	☐	MRI-based neuroimaging
